# Interferon regulatory factor 3 plays an anti-inflammatory role in microglia by activating the PI3K/Akt pathway

**DOI:** 10.1186/1742-2094-8-187

**Published:** 2011-12-30

**Authors:** Leonid Tarassishin, Hyeon-Sook Suh, Sunhee C Lee

**Affiliations:** 1 Departments of Pathology (Neuropathology), Albert Einstein College of Medicine, 1300 Morris Park Avenue, Bronx NY, USA

**Keywords:** neuroinflammation, neurodegeneration, innate immunity, human, cytokines, chemokines, antiviral genes, microarray, interferon-beta, TLR

## Abstract

**Background:**

Microglia are the principal cells involved in the innate immune response in the CNS. Activated microglia produce a number of proinflammatory cytokines implicated in neurotoxicity but they also are a major source of anti-inflammatory cytokines, antiviral proteins and growth factors. Therefore, an immune therapy aiming at suppressing the proinflammatory phenotype while enhancing the anti-inflammatory, growth promoting phenotype would be of great benefit. In the current study, we tested the hypothesis that interferon regulatory factor 3 (IRF3), a transcription factor required for the induction of IFNβ following TLR3 or TLR4 activation, is critical to the microglial phenotype change from proinflammatory to anti-inflammatory, and that this phenotype change can be greatly facilitated by IRF3 gene transfer.

**Methods:**

Cultures of primary human fetal microglia were transduced with IRF3 using recombinant adenovirus (Ad-IRF3) and subjected to microarray analysis, real-time PCR, immunoblotting and ELISA to determine inflammatory gene expression. Two different types of immune stimuli were tested, the TLR ligands, poly IC (PIC) and LPS, and the proinflammatory cytokines, IL-1/IFNγ. In addition, the role of the PI3K/Akt pathway was examined by use of a pharmacological inhibitor, LY294002.

**Results:**

Our results show that Ad-IRF3 suppressed proinflammatory genes (IL-1α, IL-1β, TNFα, IL-6, IL-8 and CXCL1) and enhanced anti-inflammatory genes (IL-1 receptor antagonist, IL-10 and IFNβ) in microglia, regardless of the cell stimuli applied. Furthermore, Ad-IRF3 activated Akt, and LY294002 reversed the effects of Ad-IRF3 on microglial inflammatory gene expression. pAkt was critical in LPS- or PIC-induced production of IL-10 and IL-1ra. Significantly, microglial IFNβ protein production was also dependent on pAkt and required both Ad-IRF3 and immunological stimuli (PIC > IL-1/IFNγ). pAkt played much less prominent and variable roles in microglial proinflammatory gene expression. This anti-inflammatory promoting role of PI3K/Akt appeared to be specific to microglia, since astrocyte proinflammatory gene expression (as well as IFNβ expression) required PI3K/Akt.

**Conclusions:**

Our results show a novel anti-inflammatory role for the PI3K/Akt signaling pathway in microglia. They further suggest that IRF3 gene therapy could facilitate the microglial phenotype switch from proinflammatory ("M1-like") to anti-inflammatory and immunomodulatory ("M2-like"), in part, by augmenting the level of pAkt.

## Background

Innate immune pathways are early responses important for pathogen control and are activated by specific receptors recognizing pathogen- or danger-associated molecular patterns [1-5]. Microglia are the key cell type involved in innate immune responses in the CNS [6-8]. The properties of microglia that contribute to this phenotype include the presence of cell surface receptors that render them highly reactive to a variety of innate and adaptive immunological stimuli [9-11]. Microglial cells bear all known TLRs, as well as phagocytic receptors, purinergic receptors, class I and class II MHC antigens and co-stimulatory molecules. Microglia *in vivo *reacts almost immediately to the pathogen/danger signals by increased motility of their processes and by upregulating innate inflammatory gene expression. Although microglial activation has conventionally been linked to inflammation and neurotoxicity (M1, "classically" activated macrophage phenotype), we now know that microglial activation does not always lead to neurodegeneration, as microglia can also generate neuronal growth factors, as well as anti-inflammatory cytokines (M2, "alternatively" activated macrophage phenotype) contributing to neuroprotection [6,12,13]. In addition to microglia, astrocytes can also participate in the CNS innate inflammatory response including antiviral immunity [14]. Studies also indicate that neurons *in vivo *and *in vitro *possess pattern recognition receptors, and can respond to dsRNA by activation of the innate immune signaling pathways including the production of IFNβ [15].

Interferon regulatory factor 3 (IRF3) is a 53 kDa transcription factor crucial in the non-MyD88, TRIF pathway of TLR signaling following activation of the TLR3 or TLR4 [16-19]. Phosphorylation of critical C-terminal serine residues represents the single most important physiological mechanism of activating IRF3. Following phosphorylation, IRF3 dimerizes and translocates to the nucleus, where DNA binding and transcriptional activation of target genes occur. In addition to TLRs, IRF3 is also activated by the cytosolic dsRNA receptors (RIG-I-like receptors), which constitute the primary receptors utilized by most viruses. IRF3 activated by various receptors, in concert with NF-κB and the MAP kinases, transactivates the IFNβ gene, as well as several additional primary IRF3-dependent genes such as IP-10 (CXCL10), Rantes (CCL5), IFN-stimulated gene 56 (ISG56, aka IFN-induced protein with tetratricopeptide repeats 1, IFIT1) and arginase II [18]. IFNβ then acts in an autocrine and paracrine manner to amplify the downstream cascades of ISG synthesis including IFNα. Studies *in vitro *show that IRF3 plays an indispensible role in innate antiviral immunity including in microglia and astrocytes [14,20,21]. In addition, IRF3 is critical in neuroprotection mediated by LPS preconditioning [22], as well as in limiting injury in experimental autoimmune encephalomyelitis, an animal model of multiple sclerosis. IRF3 is also implicated as a tumor suppressor gene [23].

Despite many known biological functions of IRF3, little is known about the regulation of expression of IRF3 under normal or pathological conditions. Most cells constitutively express IRF3 *in vitro*, but whether the amount is sufficient to trigger effective antiviral or immunoregulatory function is not known. Our immunohistochemistry study demonstrates that IRF3 expression is highly cell type-specific, with most epithelial cells showing high levels of expression and mesodermally-derived cells showing low levels of expression. In the CNS, IRF3 expression is detectable in ependymal cells and choroid plexus, with little or no expression in the brain parenchyma (Tarassishin et al., Antiviral and anti-inflammatory mechanisms of the innate immune transcription factor IRF3, submitted). In Sendai virus- or HIV-infected cells *in vitro*, IRF3 can undergo proteasomal degradation, a mechanism adopted by virus to avoid cellular antiviral responses [24,25]. In the current study, we used primary human microglial cells in culture to test the hypothesis that IRF3 is a critical regulator of microglial cytokine and chemokine expression and that increasing microglial IRF3 protein expression by adenovirus-mediated gene transfer can alter the microglial activation phenotype from proinflammatory to anti-inflammatory or immunoregulatory, which we termed "M1-like" and "M2-like", respectively (see Discussion).

## Methods

### Microglial culture

Human CNS cell cultures were prepared from human fetal abortuses as described with minor modifications [26]. All tissue collection was approved by the Albert Einstein College of Medicine Institutional Review Board. Written consent was obtained from the participants of the study. A copy of the consent is available for review by the Editor-in-Chief of this journal. Primary mixed CNS cultures were prepared by enzymatic and mechanical dissociation of the cerebral tissue followed by filtration through nylon meshes of 230- and 130-μpore sizes. Single cell suspension was plated at 1-10 × 10^6 ^cells per ml in DMEM (Cellgro, now ThermoFisher Scientific) supplemented with 10% FBS (Gemini Bio-products, Woodland, CA), penicillin (100 U/ml), streptomycin (100 μg/ml) and fungizone (0.25 μg/ml) (complete medium) for 2 weeks, and then microglial cells were collected by aspiration of the culture medium. Monolayers of microglia were prepared in 60-mm tissue culture dishes at 1 × 10^6 ^cells per 3 ml medium or in 96-well tissue culture plates at 4 × 10^4 ^per 0.1 ml medium. Four to eighteen hours later, cultures were washed to remove non-adherent cells (neurons and astrocytes). Microglial cultures were highly pure consisting of > 98% CD68^+ ^cells.

### Adenoviral vectors

Ad-IRF3 was created with pCMV-BL wildtype IRF3 plasmid (gift of John Hiscott, McGill University, Montreal) and human serotype 5 recombinant adenovirus (Adeno-X expression System 1) from BD Biosciences following the manufacturer's protocol. IRF3 wild-type (WT) IRF3-expressing adenovirus was constructed by first excising from pCMV-BL cDNA corresponding to WT IRF3 at the *Eco*RV and *Xho*I sites. The insert was cloned into the *Eco*RV and *Xho*I sites in pBluescript, then excised using *Xba*I and *Kpn*I. cDNA was subsequently ligated into the pShuttle vector (BD Biosciences). cDNA was excised according to the manufacturer's instructions with PI-*Sce*I and I-*Ceu*I, then cloned into the BD-AdenoX vector. A *Pac*I-digested linear piece of DNA containing the cDNA of WT IRF3 along with the adenovirus genome was transfected into HEK293 cells. At later times, supernatants were tested for production of recombinant adenovirus and expanded in culture. Ad-IRF3 does not contain a reporter gene. Adenovirus containing the GFP gene (Ad-GFP) and the lacZ gene (Ad-β-gal) were obtained from Dr. Mario Stevenson, University of Massachusetts, and Dr. Mark J. Czaja, Albert Einstein College of Medicine, respectively. All recombinant adenoviral vectors were amplified and purified using the service of the Gene Therapy Core of Albert Einstein College of Medicine.

### Adenovirus-mediated gene transfer and cell stimulation

**Figure 1:**
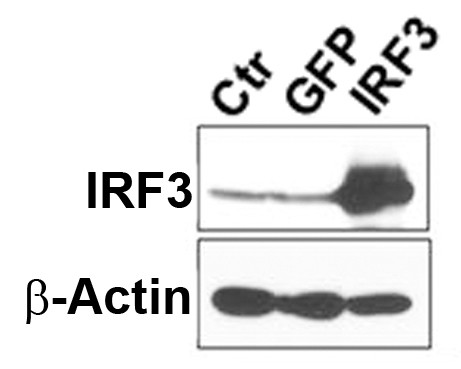
**Expression IRF3 in human microglia**. Microglial cultures were treated with medium alone (Ctr) or infected with Ad-GFP or Ad-IRF3 for 48 h, then analyzed by SDS-PAGE and western blotting for (total) IRF3, as described in the Methods.

We examined human microglia for their gene expression and cell signaling profiles following IRF3 (or control GFP or β-gal) overexpression using adenovirus-mediated gene transfer [27,28]. Cell transduction with serial dilutions of the viral vectors demonstrated that approximately 70-90% of cells were transduced after 48 h of adenoviral infection at 500 multiplicity of infection (not shown), similar to astrocytes [29]. A representative western blot analysis of IRF3 protein expression in control, Ad-GFP and Ad-IRF3 transduced microglial cultures is shown in Figure 1. Cultures that were pre-incubated with adenovirus for 48 h were then activated with cytokines (IL-1β and IFNγ) or the TLR ligands poly IC (PIC) or LPS for an additional 30 min to 72 h, as specified in individual experiments. LPS and poly IC (PIC) were purchased from Sigma-Aldrich (St. Louis, MO). Recombinant human IFN- (specific activity, 1 ng = 20 U) and IL-1β were purchased from Peprotech (Rocky Hill, NJ). Cultures were treated with PIC at 10 μg/ml, LPS at 100 ng/ml or cytokines at 10 ng/ml. For PI3K/Akt inhibition, cells were pre-treated with LY294002 at 10 μM one hour prior to cell stimulation with TLR ligands or cytokines. In all experiments, culture medium was changed a low serum medium (DMEM + 0.5% FBS) immediately before cell stimulation.

### Western blot analysis

Western blot analysis was performed as previously described [14] with minor modifications. Briefly, cell cultures in 60 mm dishes were scraped into lysis buffer (PBS plus protease inhibitors from Sigma) at various time points. Thirty to fifty micrograms of protein was separated by 10% sodium dodecyl sulfate-polyacrylamide gel electrophoresis and then transferred to polyvinylidene difluoride membrane. The blots were blocked in PBS-0.1% Tween-20 containing 5% nonfat milk and then incubated with antibodies at 4°C for 16 h. Primary antibodies were against p-Akt (Ser 473), Akt, p-ERK and p-JNK (Cell Signaling, Danvers, MA) and applied at a dilution of 1:250 for all. The secondary antibody was either horseradish peroxidase-conjugated anti-mouse or anti-rabbit IgG (Pierce Biotechnology, Rockford, IL) and was used at 1:1,000 for 1 h at room temperature (RT). Signals were developed using enhanced chemiluminescence (Pierce Biotechnology, Rockford, IL). All blots were reprobed with β-actin (Cell Signaling, Danvers, MA) to control for protein loading. Densitometric analysis was performed using ImageJ software (NIH).

### Enzyme-linked immunosorbent assay (ELISA)

IFNβ levels were determined with VeriKine-HS Human IFNβ Serum ELISA kit (sensitivity: 2.3-150 pg/ml) from PBL Interferon Source (Piscataway, NJ), according to the manufacturer's protocol. Luminex Multiplex ELISA was performed with a customized kit according to the manufacturer's protocol (Millipore Corp. Billerica, MA). IL-1β, TNFα, IL-6, IL-8, IL-10, IL-1ra and IP-10 ELISAs were performed using the antibody pairs purchased from the R&D Systems (Minneapolis, MN). Briefly, polystyrene 96-well plates (Nunc) were pre-coated overnight at RT with specific capture Ab, then blocked with 1% BSA in buffer A (PBS plus 0.1% Tween 20) for 1 h at RT. The plates were then incubated with standard cytokine dilutions or cell culture media for 2 h at RT, washed with buffer A, and incubated with the biotinylated detection Ab for 2 h at RT. After the second wash, the plates were incubated with HRP-streptavidin for 20 min at RT and washed again. The signal was developed after addition of 3,3',5,5'-tetramethylbenzidine-peroxidase EIA kit (Bio-Rad) for 4-5 min and the reaction was stopped by 1 M H_2_SO_4_. Microplate reader (Dynex Technologies) was used to detect the signals with 450 nm and correction at 530 nm. The samples were diluted until the values fell within the linear range of each ELISA detection.

### Real-time PCR

Quantitative real-time reverse transcription-PCR (Q-PCR) was performed as described previously [14,27,29]. Initial microglial experiments including both porphobilinogen deaminase (PBDA) and GAPDH as housekeeping genes showed that the results were very similar with either gene as a control. Therefore, all subsequent experiments were done with PBDA and all results were calculated using PBDA as a control. Total RNA was extracted with TRIzol (Invitrogen Life Technologies), following the manufacturer's instructions. PCR was performed using a SYBR green PCR mix and conducted with the ABI Prism 7900HT (Applied Biosystems). All values were expressed as the increase relative to the expression of PBDA. The median value of the replicates for each sample was calculated and expressed as the cycle threshold (*C_T_*; cycle number at which each PCR reaches a predetermined fluorescence threshold, set within the linear range of all reactions). Δ*C_T _*was calculated as *C_T _*of endogenous control gene (PBDA) minus *C_T _*of target gene in each sample. The relative amount of target gene expression in each sample was then calculated as 2^Δ*CT*^. Fold change was calculated by dividing the value (2^Δ*CT*^) of test sample by the value (2^Δ*CT *^) of control sample (control = 1). TaqMan PCR was performed with miR-155 primers according to the manufacturer's protocol (Applied Biosystems).

### Microarray analysis

Highly enriched microglial cultures were subjected to microarray analysis using the Illumina platform. Briefly, for each total RNA sample, linear amplification and biotin-labeling of total RNA (500 ng) were carried out using the Illumina TotalPrep RNA Amplification Kit (Ambion Applied Biosystems, Austin, TX). Whole-genome expression analysis was carried out by hybridization of amplified RNA to an Illumina HumanHT-12 v3 Expression BeadChip (Illumina Inc., San Diego, CA). With this beadchip, we interrogated greater than 48,000 probes per sample, targeting genes and known alternative splice variants from the RefSeq database release 17 and UniGene build 188. Controls for each RNA sample (greater than 1000 bead types) confirmed sample RNA quality, labeling reaction success, hybridization stringency, and signal generation. All expression data were quantile normalized and background-subtracted prior to analysis using BeadStudio software (Illumina Inc.).

### Statistical Analysis

For multiple comparisons, one-way ANOVA with Bonferroni post test was performed. For comparison of two groups, Student's t-test was used. Fold induction or inhibition by Ad-IRF3 from multiple experiments was compared to control (Ad-GFP) using single sample t-test. P values < 0.05 were considered significant. All statistics were performed using GraphPad Prism 5.0 software.

## Results

### Adenovirus-mediated IRF3 gene transfer alters the gene expression profile of cultured human microglia

Our previous studies have suggested that over-expression of IRF3 by adenovirus-mediated gene transfer (Ad-IRF3) might suppress microglial proinflammatory cytokine expression while increasing anti-inflammatory and antiviral gene expression [21]. In this study, we systematically examined the changes in microglial gene expression following exposure to Ad-IRF3.

Cultures of primary human fetal microglia were infected with recombinant Ad-IRF3 or the control adenovirus (Ad-GFP or Ad-β-gal) for 48 h as previously described, and then further treated with inflammatory stimuli (LPS, PIC or IL-1β/IFNγ) for an additional 6 h - 24 h. Gene expression was examined by microarray analysis with the Illumina HumanHT-12 v3 Expression BeadChip, or by real-time PCR, and protein expression was examined by ELISA.

Representative data from microarray analyses are shown in Table 1 for PIC-stimulated microglia and Table 2 for IL-1/IFNγ-stimulated microglia. Entire microarray data sets are available as Supplementary Material (Additional Files 1 and 2). In PIC-treated cultures, IRF3-enhanced genes included IFNβ, IL-29 (IFNλ, "type III IFN") [30,31], IRF7, an inducible transcription factor which synergizes with IRF3 [21,32], many ISGs (not shown, see Supplemental Materials), TLR7, a TLR shown to mediate antiviral and anti-inflammatory functions in myeloid cells [33], and IL-10 receptor. Intriguingly, IL-1α and IL-1ra, as well as the IL-12 family cytokines IL-23 and IL-27 [34] were differentially regulated, showing increase in IL-1ra and IL-27 and decrease in IL-1α/β and IL-23 (Table 1). These results suggest that Ad-IRF3 can suppress the Th1/Th17 activation pathway and promote the Th2 pathway in microglia.

**Table 1:** Microarray studies of microglia transduced with Ad-IRF3 or Ad-β-gal and stimulated with poly IC (PIC):

*Gene*	*Ratio* *(IRF3/β-gal)*	*Difference in expression* *(IRF3 minus β-gal)*
*IFNB*(IFNβ)	61.0	3863
*IRF7*	2.3	702
***IL-1A *(IL-1α)**	**0.22**	**-1413**
*IL-1RN *(IL-1ra)	2.3	10836
*IL-27*	7.2	1291
*IL-29 *(IFN lambda)	75.0	231
*IL-10RA *(IL-10Rα)	3.3	954
***IL-23 *(p19)**	**0.47**	**-372**
*TLR7*	2.2	813

Human fetal microglia were transduced with adenovirus (Ad-IRF3 or Ad-β*-*gal) and further treated with the TLR3 ligand PIC for 16 h. Microarray was performed using the Illumina platform.

**Table 2:** Microarray studies of microglia transduced with Ad-IRF3 or Ad-GFP and stimulated with IL-1/IFNγ:

*Gene*	*Ratio* *(IRF3/GFP)*	*Difference in expression* *(IRF3 minus GFP)*
*IFNB *(IFNβ)	3.5	418
*IRF7*	2.3	1441
*IFIT1*	11.2	4464
*SOCS1*	2.6	776
***IL-1A *(IL-1α)**	**0.2**	**-708**
***IL-1B *(IL-1β)**	**0.1**	**-4738**
*IL-1RN *(IL-1ra)	6.4	2211
*IL-10*	1.9	364
*IL-10RA *(IL-10R)	2.4	1145
*IL-27*	1.8	250
***IL-8***	**0.3**	**-15409**
***CXCL1***	**0.5**	**-167**

Human fetal microglia were transduced with adenovirus for 2 days and further treated with IL-1/IFNγ for 16 h. Microarray was performed using the Illumina platform.

Similar trends were observed in IL-1/IFNγ-treated microglial cultures (Table 2), i.e., downregulation of proinflammatory cytokine genes such as IL-1α, IL-1β, IL-8 and CXCL1 (GROα), but upregulation of anti-inflammatory genes, antiviral genes and ISGs, such as IL-1ra, IL-10, IL-10 receptor, IFNβ, IFIT1, IRF7, suppressor of cytokine signaling 1 (SOCS1) and IL-27 (Table 2).

The microarray data show that microglial inflammatory and antiviral genes are differentially regulated in the presence of increased IRF3 protein expression, and that the responses are similar regardless of the stimuli used (TLR ligands vs. proinflammatory cytokines).

### Q-PCR validation of the Ad-IRF3 effects in microglial inflammatory gene expression

**Figure 2:**
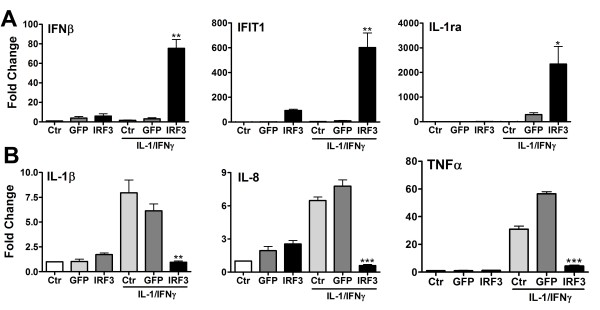
**Microglial genes that are upregulated (A) or downregulated (B) by Ad-IRF3 (Q-PCR)**. Primary human microglial cultures were exposed to medium alone (Ctr), Ad-GFP (GFP) or Ad-IRF3 (IRF3) at 500 multiplicity of infection (moi) for 48 h, then stimulated with IL-1β and IFNγ at 10 ng/ml each for 16 h. Q-PCR analysis was performed using PBDA as a control. Y-axis denote fold changes calculated as described in the Methods section, then normalized to the control level (without cytokine treatment). Note varying Y-axis scales. All samples were tested in triplicate, and p-values were calculated by t-test (GFP vs. IRF3) using GraphPad Prism 5. * p < 0.05, ** p < 0.01, *** p < 0.001.

We also employed Q-PCR to examine microglial gene expression following exposure to Ad-IRF3. Figure 2 shows a typical experiment in which microglial cultures derived from a single case were tested in six different conditions: uninfected (Ctr), Ad-GFP-infected or Ad-IRF3-infected, each with or without treatment with IL-1/IFNγ. Selected genes were examined based on the microarray data, and the results showed that antiviral and anti-inflammatory genes such as IFNβ, IFIT1 and IL-1ra were robustly upregulated by Ad-IRF3, and proinflammatory genes such as IL-1β, IL-8 and TNFα were downregulated by Ad-IRF3.

### Q-PCR validation of the Ad-IRF3 effects in multiple microglial cases

**Figure 3:**
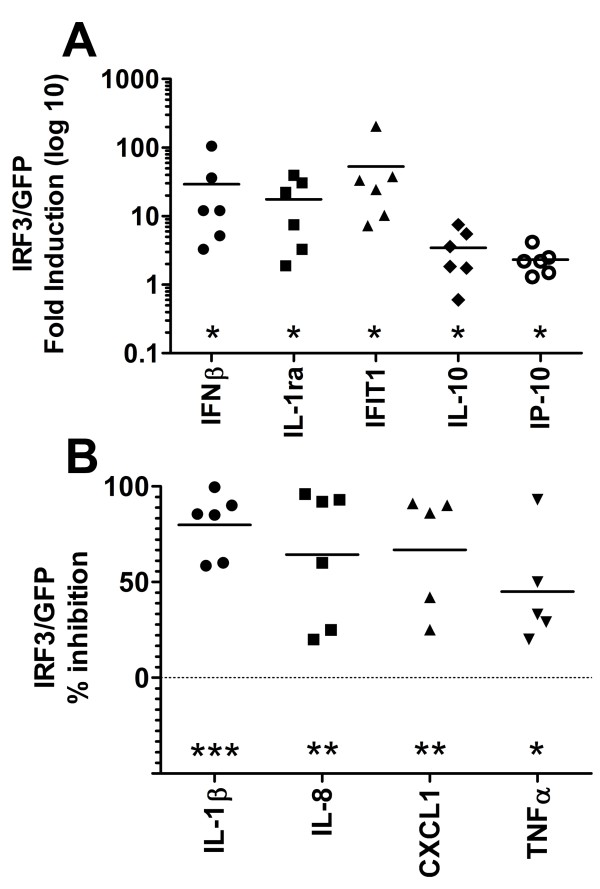
**Q-PCR validation of the Ad-IRF3 effects in multiple microglial cases**. Microglial cultures were transduced with Ad-GFP or Ad-IRF3, treated with IL-1β/IFNγ, and then Q-PCR analysis was performed as described in Figure 2 legend. Data were generated from 5-6 different microglial cases, with each symbol representing a different case. **(A) **Genes upregulated by IRF3: fold changes in mRNA were calculated as described in the Methods. Fold induction by Ad-IRF3 over Ad-GFP was expressed in a log scale (0.1 = no induction). **(B) **Genes downregulated by IRF3. Y-axis denotes % inhibition by IRF3 calculated using the formula, 100 × [1 - Ad-IRF3/Ad-GFP]. In both A and B, p-values were generated using single sample t-test (GraphPad Prism 5): * p < 0.05, ** p < 0.01, *** p < 0.001.

We have analyzed data from microglial cultures derived from multiple donors in order to determine whether the Ad-IRF3 effects are significant across many cases. Q-PCR data were compiled from several microglial cases treated with IL-1/IFNγ and grouped into significantly upregulated and downregulated genes, based on single sample t-test. Ad-IRF3-upregulated genes are shown in Figure 3A as ratios of gene expression in Ad-IRF3 culture to Ad-GFP culture in a log 10 scale. Ad-IRF3-downregulated genes are shown in Figure 3B as % inhibition, calculated by 100 × [1 - Ad-IRF3/Ad-GFP]. These results once again confirm that the two groups of genes ("M1-like" and "M2-like") are differentially regulated by Ad-IRF3 in microglia.

### Ad-IRF3 effects on microglial cytokine protein production

**Figure 4:**
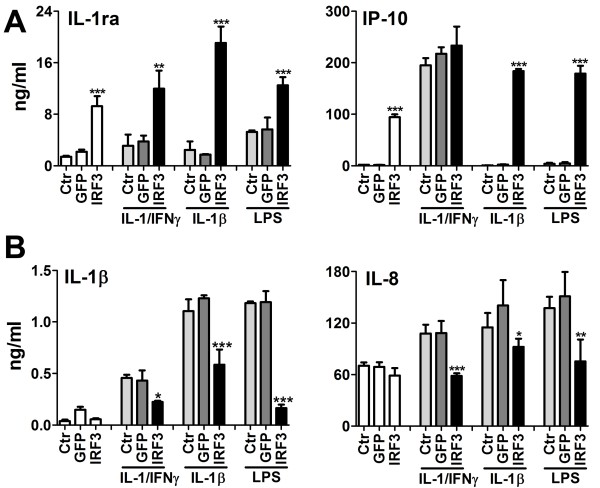
**Ad-IRF3 modulated microglial cytokine production (ELISA)**. Microglia were grown in 96-well plates and left uninfected (Ctr) or infected with Ad-GFP or Ad-IRF3 for 48 h, and treated with IL-1β/IFNγ (10 ng/ml each), IL-1β alone (10 ng/ml) or LPS (100 ng/ml) for 24 h. Culture supernatants (IL-1ra, IP-10, and IL-8) or cell lysates (IL-1β) were analyzed for cytokine levels by ELISA as described in the Methods section. All samples were tested in triplicate, and p-value was calculated by t-test (GFP vs. IRF3). * p < 0.05, ** p < 0.01, ***p < 0.001.

We next performed Luminex multiplex beads-based protein analyses of IL-1/IFNγ-stimulated microglia to determine whether the Ad-IRF3-induced mRNA changes are reflected at the protein level. We found that IFNα2 and IL-1ra were increased while IL-1α and TNFα were decreased by Ad-IRF3 (not shown). We next expanded the study to compare the responses to different stimuli (IL-1/IFNγ, IL-1 alone, and LPS) in the same microglial cases, and examined the production of IL-1β, IL-1ra, IL-8 and IP-10 by individual ELISA (Figure 4). The results show that the amounts of proinflammatory cytokines such as IL-1β and IL-8 were markedly decreased by Ad-IRF3, while the amounts of IL-1ra and IP-10 (a known IRF3-dependent gene) [18,35] were increased. These results confirm that Ad-IRF3 differentially regulates microglial cytokine production, regardless of the types of stimuli applied (TLR ligands vs. IL-1/IFNγ).

### Ad-IRF3 activates the PI3K/Akt pathway in microglia

**Figure 5:**
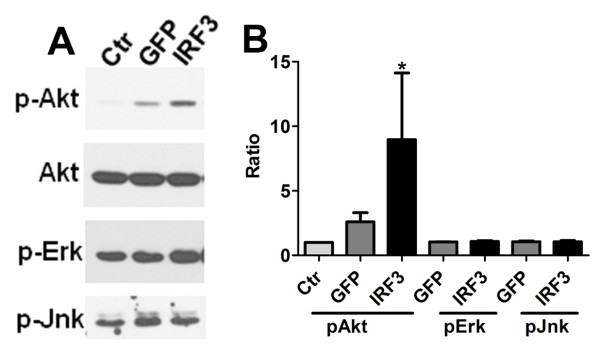
**Ad-IRF3 activates pAkt in human microglia**. (A) Microglial cultures were infected (or not) with Ad-GFP or Ad-IRF3 for 48 h, then analyzed by SDS-PAGE and western blotting for p-Akt (Ser 473), p-Erk, p-Jnk and total Akt as described in the Methods section. (B) Densitometry was performed with ImageJ and the ratios of pAkt to total Akt were calculated from 3 different microglial cases. * p < 0.05 by t-test (GFP vs. IRF3).

In order to determine the mechanism by which Ad-IRF3 mediates its effects on microglial cytokine expression, we examined cell signaling pathways altered by Ad-IRF3 by western blot analysis. Three different cases of microglial cultures were transduced with Ad-IRF3 or Ad-GFP (or neither, Ctr) for 48 h, and were subjected to western blot analysis for p-Akt, p-Erk, p-Jnk, and total Akt. Figure 5A demonstrates a representative western blot and Figure 5B demonstrates densitometric analysis normalized to the control level (Ctr = 1) from three microglial cases. The results show that the levels of p-Akt (Ser 473) increased in the presence of Ad-IRF3, whereas those of p-Erk or p-Jnk were unchanged.

### Role of the PI3K/Akt pathway in Ad-IRF3-mediated modulation of microglial gene expression

**Figure 6:**
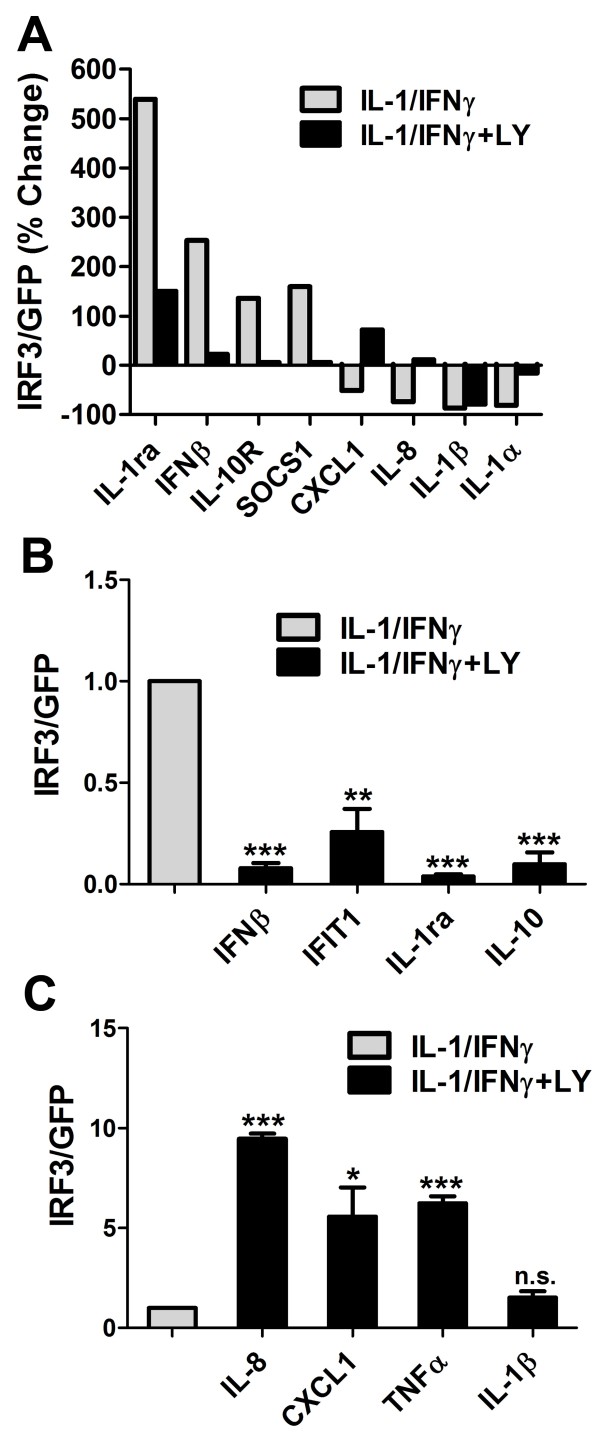
**The effect of the PI3K/Akt inhibitor on Ad-IRF3-mediated changes in microglial gene expression**. Microglial cells were left uninfected (Ctr) or infected with Ad-GFP or Ad-IRF3 for 48 h. The cells were then pre-treated with LY294002 (10 μM) for 1 h followed by stimulation with IL-1β and IFNγ for an additional 16 h. **(A) **Microarray analysis was performed using the Illumina platform, and the data are presented as % change (Ad-IRF3 over Ad-GFP) as described in the Results section. The data show that LY294002 reversed the effect of Ad-IRF3 in microglial inflammatory gene expression, showing suppression of IRF3-enhanced genes and enhancement of IRF3-suppressed genes. **(B, C) **Q-PCR confirmation of the microarray data (B: IRF3-enhanced genes, C: IRF3-suppressed genes). Q-PCR analysis was performed using PBDA as an internal control as described in Methods. At least 3 different cases were tested in each experiment. All data were normalized to the level in Ad-GFP culture (= 1).

In order to determine whether pAkt contributed to Ad-IRF3-mediated modulation of microglial gene expression, we employed a pharmacological inhibitor of PI3K, LY294002. Microglial cultures were transduced with Ad-IRF3 or Ad-GFP then stimulated with IL-1/IFNγ in the presence or absence of LY294002, as described in the Methods. The results were examined by microarray (Figure 6A) and also by Q-PCR (Figure 6B and 6C). In Figure 6A, gene expression ratios (Ad-IRF3 vs. Ad-GFP) were expressed as % change, in which "0" represents no change (IRF3/GFP = 1), + 100% represents two-fold increase (IRF3/GFP = 2), and -50% represents 50% inhibition (IRF3/GFP = 0.5). The results showed that the PI3K inhibitor exhibited differential effects on the expression of the two groups of genes, i.e., suppression of Ad-IRF3-induced genes (IL-1ra, IFNβ, IL-10R and SOCS1) and increase of Ad-IRF3-inhibited genes (CXCL1, IL-8, IL-1α, and IL-1β). The complete microarray data set is available as Supplemental Material (Additional File 2).

These results are validated by Q-PCR. Figure 6B and 6C demonstrate Q-PCR data derived from several microglial cases, shown as normalized values (cytokines alone = 1). They confirm that LY inhibited Ad-IRF3-upregulated genes (IFNβ, IFIT1, IL-1ra and IL-10) while increasing Ad-IRF3-inhibited genes (IL-8, CXCL1, TNFα, and IL-1β). However, the effect of LY on IL-1β mRNA expression was not significant, reflecting the results obtained with microarray (see Figure 6A). Taken together, these results demonstrate that the PI3K/Akt pathway significantly contributes to the differential gene regulation induced by Ad-IRF3 in microglia.

### The role of the PI3K/Akt pathway in microglial inflammatory gene expression

**Figure 7:**
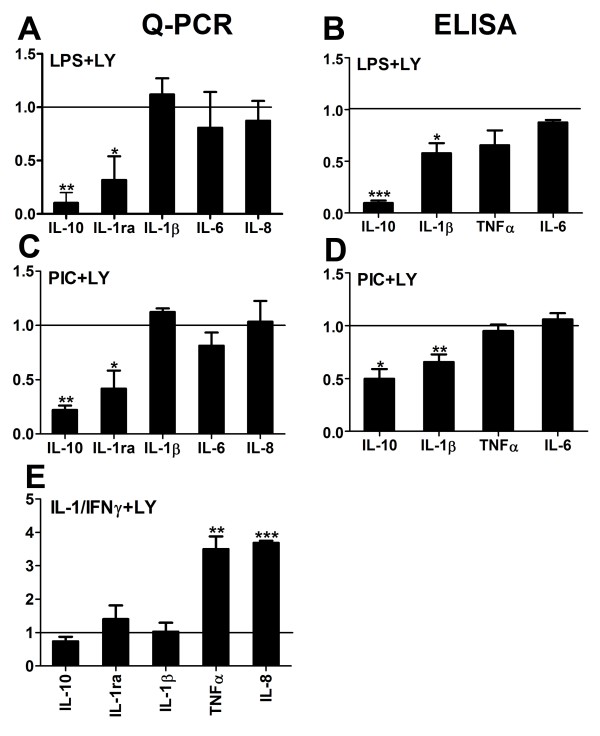
**The effect of LY294002 on TLR3/4- or IL-1/IFNγ-induced microglial inflammatory gene expression**. Microglial cells were pre-treated with LY294002 for 1 h and then stimulated with LPS (100 ng/ml), PIC (10 μg/ml), or IL-1β and IFNγ (10 ng/ml) for 16 h. Q-PCR (left column: A, C and E) and ELISA (right column: B and D) were performed as described above. All values were normalized to those obtained in cultures stimulated with LPS, PIC or IL-1/IFNγ *without *LY294002 (= 1). At least 3 cases were analyzed for each experiment, and statistics were performed using t-test comparing values with vs. without LY294002.

Because our data suggest a major role of PI3K/Akt in Ad-IRF3-mediated immune modulation, we next examined the effect of LY294002 on microglial cytokine gene induction by TLR activation or IL-1/IFNγ. Microglia were stimulated with LPS, PIC or IL-1/IFNγ in the presence or absence of LY294002 and the expression of selected cytokine genes was examined by Q-PCR and ELISA. Shown in Figure 7 are results from multiple microglial cases, normalized to the values induced by LPS, PIC or IL-1/IFNγ alone. They show that the PI3K/Akt pathway is involved in LPS- or PIC-mediated induction of anti-inflammatory cytokine genes (IL-10 and IL-1ra), but that it had little or no effect on proinflammatory cytokine mRNA expression (IL-1β, TNFα, IL-6 and IL-8). Interestingly, LY294002 suppressed IL-1β protein production, although it had no significant effect on IL-1β mRNA. As noted before [21], human microglia responded remarkably similarly to LPS or PIC. The effects of LY294002 on cytokines induced by IL-1/IFNγ were different from those observed using TLR ligands (Figure 7A and 7B). LY294002 had no significant effects on anti-inflammatory gene expression, but it had significant *stimulatory *effects on proinflammatory gene expression (TNFα, IL-8), with no change in IL-1β mRNA levels (see below for ELISA data).

**Figure 8:**
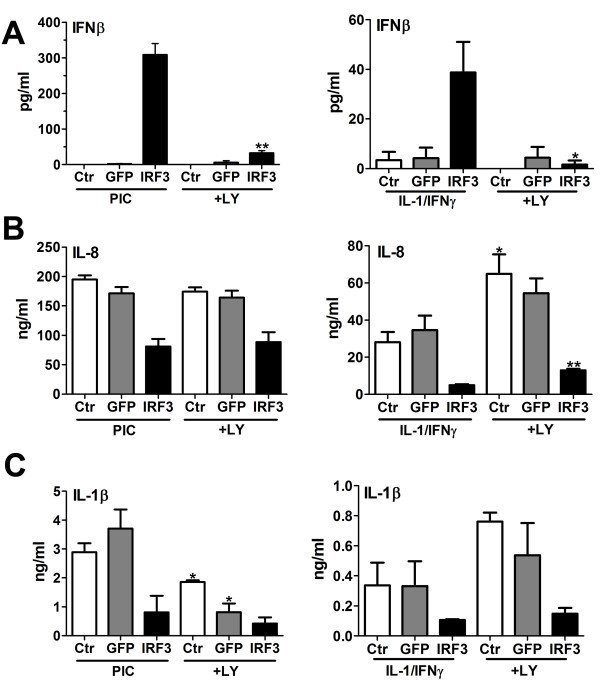
**The effect of LY294002 on PIC-, IL-1/IFNγ-, or Ad-IRF3-induced microglial cytokine expression (ELISA)**. Microglia cultures were grown in 96-well plates, infected (or not) with Ad-GFP or Ad-IRF3 for 48 h. Cultures were then pretreated with LY (10 μM) for 1 h before stimulation with PIC (10 μg/ml) or IL-1β/IFNγ (10 ng/ml each) for an additional 24 h. Culture supernatants or cell lysates were analyzed for IFNβ (**A**), IL-8 (**B**) and IL-1β (**C**) by ELISA as described in the Methods section. All samples were tested in triplicates, and p-values was determined by t-test (*with vs. without *LY294002). * p < 0.05, ** p < 0.01.

Since these data suggest a possible stimulus-dependent role of PI3K in microglial inflammatory gene induction, we next compared PIC and IL-1/IFNγ as stimuli in the same microglial case. The role of Ad-IRF3 was also determined. Microglia were transduced with Ad-IRF3 or Ad-GFP (or neither) and further stimulated with PIC or IL-1/IFNγ in the presence or absence of LY294002. The production of IFNβ, IL-8 and IL-1β was determined by ELISA (Figure 8).

Measurement of IFNβ using a highly sensitive ELISA kit (detection range 2.3 - 150 pg/ml) demonstrated that neither PIC nor IL-1/IFNγ (without Ad-IRF3) induced detectable amounts of IFNβ from microglia (Figure 8A). IFNβ was produced when cells were exposed to both Ad-IRF3 and immune stimuli (PIC >> IL-1/IFNγ, note different Y-axis scales). Furthermore, IFNβ production was almost completely inhibited by LY294002. In contrast, LY294002 had no effect on PIC-induced IL-8 protein production (similar to mRNA data in Figure 7C), but it increased IL-8 production by IL-1/IFNγ (similar to mRNA data in Figure 7E), suggesting a suppressive role of PI3K/Akt in IL-1/IFNγ-induced IL-8 expression (Figure 8B). Furthermore, LY294002 suppressed PIC-induced IL-1β protein production (similar to data in Figure 7D), but it increased IL-1/IFNγ-induced IL-1β protein production (similar to its effect on IL-8 protein). The effect of LY294002 in the presence of Ad-IRF3 resembled the results obtained by microarray and Q-PCR in Figure 6. For all three cytokines, PIC provided a stronger stimulus than IL-1/IFNγ for microglia (Figure 8, note different Y-axis scales).

Together, our experiments with LY294002 show that the PI3K/Akt pathway plays a crucial role in the induction of key anti-inflammatory and immunomodulatory genes such as IL-1ra, IL-10 and IFNβ from microglia. They also show that boosting the amount of IRF3 protein in microglia is necessary for adequate IFNβ response upon further stimulation with TLR ligands or cytokines. The PI3K/Akt pathway plays dual roles in proinflammatory cytokine production from microglia, depending on the nature of the stimuli used to induce cytokines: it plays a suppressive role when cytokines (IL-1/IFNγ) are used as inducing stimuli, but shows little effects when the TLR3/4 ligands (LPS or PIC) are used as stimuli. One exception was TLR3/4-induced IL-1β protein expression, which was enhanced by PI3K/Akt presumably by post-transcriptional modification, since mRNA levels did not change.

### Role of PI3K/Akt in astrocyte cytokine production

**Figure 9:**
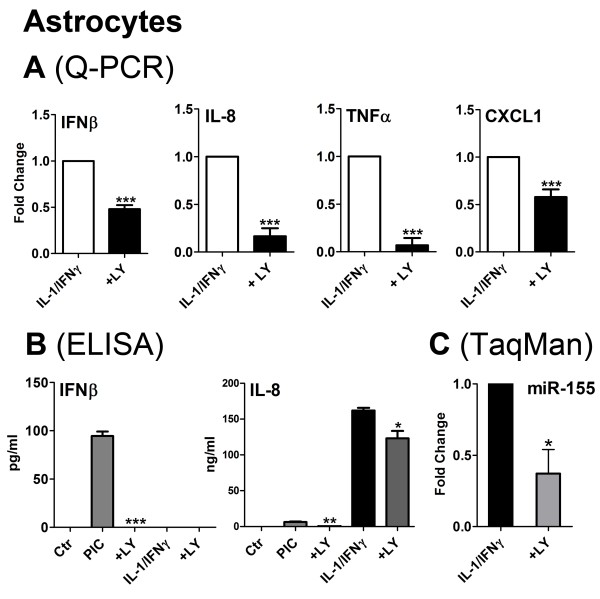
**The effect of the PI3K pathway inhibitor LY294002 on astrocyte cytokine and chemokine expression**. Primary human fetal astrocytes were stimulated with IL-1/IFNγ or PIC, with or without LY294002, essentially in the same manner described for microglia. Q-PCR (**A**) or ELISA (**B**) was performed to determine the expression of "proinflammatory" (IL-8, TNFα, and CXCL1) genes or IFNβ gene. (**C**) TaqMan Q-PCR was performed to determine the expression of microRNA, miR-155. All values were normalized to those without LY294002 and t-test was used to determine p values. * p < 0.05, ** p < 0.01, *** p < 0.001.

In order to determine whether the "anti-inflammatory" role of pAkt was unique to microglia, we examined astrocyte responses to LY294002. Primary human fetal astrocytes were prepared and stimulated as previously described [14,26,29]. The cultures were stimulated IL-1/IFNγ or PIC, with or without LY294002, essentially in the same manner described for microglia. Q-PCR or ELISA was performed to determine the expression of "proinflammatory" (IL-8, TNFα, and CXCL1) genes or IFNβ gene. TaqMan Q-PCR was performed to determine the expression of microRNA, miR-155, as described [29]. The results show that PI3K has a very different role in astrocytes, as LY294002 suppresses all proinflammatory genes, IFNβ, as well as the proinflammatory microRNA, miR-155 (Figure 9). These results are consistent with the role of PI3K/Akt upstream of NF-κB or MAPK in the astrocyte signal transduction cascades.

### Summary and hypothesis

**Figure 10:**
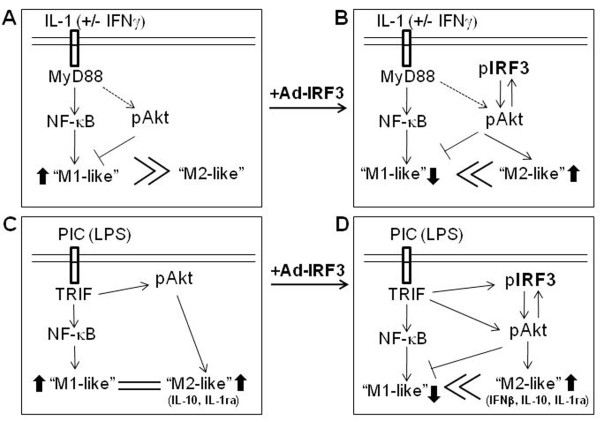
**Schematic representation of the proposed molecular events that are affected by Ad-IRF3 in microglia**. Ad-IRF3, through suppression of "M1-like" gene expression and over-expression of "M2-like" gene (IFNβ, IL-10 and IL-1ra) expression in microglia, can suppress proinflammatory cytokine cascades that are implicated in neurodegeneration. pAkt plays a major role in regulating microglial inflammatory gene expression. In Ad-IRF3-transduced microglia, a positive feedback mechanism between pAkt and pIRF3 could induce a state that is dominated by the "M2-like" cytokines. See text for details.

Our results show that the PI3K/Akt pathway plays a crucial role in the induction of key cytokines of anti-inflammatory and immunomodulatory nature from microglia (i.e., IL-1ra, IL-10 and IFNβ), regardless of the stimuli applied (Figure 10). (A) In IL-1/IFNγ-stimulated microglia, while large amounts of proinflammatory cytokines are produced, little or no anti-inflammatory or immunoregulatory cytokines are produced. The PI3K/Akt pathway functions as an endogenous inhibitor of proinflammatory gene expression, possibly by suppressing proinflammatory factors such as miR-155. (B) Transduction of microglia with Ad-IRF3 robustly increases the production of anti-inflammatory and immunoregulatory genes upon stimulation with IL-1/IFNγ, while decreasing the production of proinflammatory genes. This effect is presumably mediated by increased activation of Akt by Ad-IRF3. (C, D) In TLR3/4-activated microglia, Akt is activated downstream of TRIF, which critically contributes to the induction of anti-inflammatory and immunoregulatory genes such as IFNβ [36]. However, in normal microglia, the low amount of IRF3 protein precludes effective IFNβ production (C). Following transduction with Ad-IRF3, a positive feedback loop between pAkt and pIRF3 becomes established which then amplifies induction of anti-inflammatory and immunoregulatory genes and suppression of proinflammatory genes through multiple mechanisms (D). For simplicity, we refer to the two phenotypes of microglia as "M1-like" and "M2-like", respectively (see Discussion).

## Discussion

Our study was designed to investigate the role of IRF3 transgene expression in microglial inflammatory activation. Our data in primary human microglial cultures show that adenovirus-mediated IRF3 transgene expression changes the microglial cytokine profile from a proinflammatory phenotype to an anti-inflammatory or immunoregulatory phenotype. Specifically, the expression of IL-1ra, IL-10 and IFNβ was markedly induced, while the expression of many proinflammatory cytokines such as IL-1 was suppressed consistently and significantly. Additional suppressed proinflammatory genes included TNFα, IL-6 and IL-8 and CXCL1.

We refer to the microglial cytokine expression profile changes described here as "M1-like" or "M2-like", following the general scheme of M1 and M2 activation phenotypes developed in mouse macrophages and subsequently adopted to describe microglial activation phenotypes [6]. There are a number of differences between human microglia and murine microglia. For example, although iNOS is a prototypic marker of M1-activated murine microglia, it is not expressed by human microglia [37]. In addition, human microglia do not express certain Th1 or Th2 cytokines such as IFNγ or IL-4. There might also be additional differences between macrophages and microglia. For these and other reasons, we refer to the microglial phenotypes described here as "M1-like" or "M2-like".

Importantly, we note these changes regardless of the types of immunological stimuli applied (TLR3/4 ligands as well as IL-1/IFNγ, to mimic non-infectious neuroinflammatory conditions). The observed effects of IRF3 transgene in the suppression of proinflammatory cytokine genes is novel and points to a mechanism by which IRF3 influences other signaling pathways. In addition, we have obtained novel findings that indicate that the PI3K pathway plays a predominantly anti-inflammatory role in microglial activation. It played a particularly potent role in the induction of anti-inflammatory and immunoregulatory cytokines such as IL-10, IL-1ra and IFNβ. These results together suggest that activation of the PI3K/Akt pathway (via Ad-IRF3, for example) in microglia can lead to the resolution of inflammation and promotion of repair under neuroinflammatory conditions [38-40].

The PI3K/Akt pathway is unique for its multitudes of roles in transcriptional regulation of cytokine genes. Employing a pharmacological inhibitor, we show that the PI3K/Akt pathway is involved in both the suppression (of "M1-like") and the enhancement (of "M2-like") of cytokine genes in IRF3-transduced microglia. One might speculate that the impressive amounts of suppression of proinflammatory genes in Ad-IRF3-transduced cells are at least in part secondary to the induction of anti-inflammatory and immunoregulatory genes, as IL-1ra, IL-10 and IFNβ each can function as a suppressor of proinflammatory cytokine expression. For example, we have previously shown that recombinant IFNβ suppresses IL-1 and increases IL-1ra production in human microglia [41]. IFNβ also induces certain chemokines [42]. Microarray analysis of human peripheral blood mononuclear cells (PBMCs) exposed to IFNβ demonstrated that distinct sets of genes are upregulated or downregulated by IFNβ, the latter including IL-1β, CXCL1, and IL-8 [43]. Therefore, IFNβ most certainly played a role as an intermediary cytokine that mediated the effect of Ad-IRF3 in our system. Additional cytokines that might have played a role in our system include IFNα, as well as type III IFNs. Type III IFNs are newly discovered interferons that share a number of similarities with type I IFNs including their mechanism of induction and their biological activities [30]. One might also speculate that the opposite effects of LY294002 on the two groups of genes can be best (and most simply) explained by the prominent role played by PI3K/Akt on microglial "M2-like" cytokine induction. Furthermore, we show that PI3K/Akt might play a different role in proinflammatory gene expression (without Ad-IRF3) depending on the stimulus applied, as that induced by IL-1/IFNγ was suppressed by PI3K/Akt, while little changes were noted in PIC-stimulated microglia, and PIC-induced IL-1β production was even increased. We also note that although IL-1 expression was consistently and potently suppressed by Ad-IRF3 transduction in microglia, its expression appeared to be least affected by the PI3K inhibitor. Therefore, multiple mechanisms must exist that mediate the effects of Ad-IRF3 on microglial cytokine expression. Additionally, the adenoviral vector may have evoked some elements of inflammatory activation in microglia and that this may have created conditions that contributed to the effects seen 48 h after adenovirus infection. Our results with LY294002 are reminiscent of those obtained in mouse macrophages deficient in phosphatase and tensin homologue (PTEN), a negative regulator of Akt, which showed similar differential regulation of cytokines, i.e., decrease in TNFα/IL-6 and increase in IL-10 [44] supporting the dual role played by PI3K/Akt in Ad-IRF3-transduced microglial cytokine expression. Our results demonstrating a pivotal role of pAkt in IFNβ production is also in line with another study of murine macrophages which demonstrated a critical role of pAkt in TLR-induced IRF3 activation and IFNβ expression downstream of TRIF signaling [36]. The anti-inflammatory role of Akt in mouse macrophages has been most convincingly demonstrated in a study in which Akt1-deficient mice injected with LPS showed increases in proinflammatory cytokine production compared to wildtype mice [45]. In the latter study, the effect of Akt1 was attributed in part to its suppression of microRNA-155 (miR-155) expression. miR-155 is a proinflammatory microRNA that increases cytokine production by targeting specific mRNAs such as suppressor of cytokine signaling (SOCS1) mRNA [29,46,47]. These results are interesting, since miR-155 was significantly elevated by IL-1/IFNγ in human microglia (data not shown), suggesting that suppression of miR-155 may be the mechanism by which Akt modulated "M1-like" cytokines in IL-1/IFNγ-stimulated microglia (Figure 10A).

The role of the PI3K/Akt pathway in cytokine production is also cell-type specific. In human astrocytes, we see that LY294002 *suppresses *both "M1-like" (TNFα and IL-8, for example) and "M2-like" cytokine (IFNβ) expression induced by PIC or IL-1/IFNγ (Figure 9). These results suggest that in astrocytes, Akt is activated upstream of NF-κB (and MAPK) following activation of TLR3 or IL-1R. In addition, LY294002 suppresses miR-155 expression in astrocytes, indicating a *positive *role for PI3K/Akt in miR-155 expression in astrocytes (Figure 9). These results demonstrate that the PI3K/Akt pathway plays a fundamentally different role in the inflammatory activation of the two glial cell types (astrocytes vs. microglia). It is also possible that microglia and astrocytes express different combinations of Akt isoforms, with each isoform having distinct immune regulatory functions. These are some of the topics that need to be explored in future studies.

Our results suggest that in Ad-IRF3-transduced microglia, a positive feed forward loop between Akt and IRF3 might be established resulting in downmodulation of inflammatory activation. For example, evidence supports that signaling through TRIF (TLR3 or TLR4) or MyD88 (TLR4 or IL-1R) activates Akt [21] that is critical in the activation of IRF3 [28,36,48,49]. Furthermore, Ad-IRF3 increases the level of pAkt, likely contributing to increased activation of IRF3, in addition to increase in total IRF3 (Figures 5 and 10). It is unclear how Ad-IRF3 increases pAkt in microglia. We do not believe this was mediated by IFNβ because we do not see measurable IFNβ in cultures treated with Ad-IRF3 alone (not shown). In addition, our previous studies showed that while IFNβ activates microglial NF-κB and MAP kinases (ERK) immediately, IFNβ (or IFNγ) does not activate Akt until later time points (6 h), indicating an indirect mechanism of activation [21,50].

The major change that we see in IRF3-transduced microglia is downmodulation of the IL-1 axis. IL-1 is a non-redundant cytokine expressed primarily by microglia and macrophages but also by T cells. Microglial IL-1 is induced early after CNS insult and is capable of activating downstream cytokine cascades, as well as auto-amplification cascades [41,51]. *In vitro*, microglial IL-1 is induced by diverse types of stimuli [4,52] and serves as a potent neurotoxin [53,54]. IL-1 is also crucial in the Th17 differentiation of human T cells [55-57]. The amount of IL-1 signal transduction is primarily determined by the relative abundance of the agonists (IL-1α or IL-1β) and the antagonist (IL-1ra). The importance of IL-1ra in human biology has been elucidated in recent discovery of an inflammatory disease caused by homozygous deletion/mutations of the *IL1RN *locus [58-60]. A term DIRA (deficiency of the IL-1ra) has been proposed to denote this life-threatening autoinflammatory disease caused by unopposed action of IL-1. Of interest, IFNβ and glatiramer acetate, disease-modifying treatments for multiple sclerosis, are both known to exert opposing effects on IL-1α/β and IL-1ra [41,61]. Therefore, the combined effects of IL-1 receptor antagonism and the robust increase in IL-10 and IFNβ production in Ad-IRF3-transduced microglia could significantly alter the neuroimmune environment in favor of resolution of inflammation and promotion of repair. The data obtained in this study should be useful in future development of therapeutic strategies aiming at neuroinflammation.

## Conclusions

In this study, we tested the hypothesis that upregulation of IRF3 protein in primary human microglia by virus-induced gene transfer could alter the microglial inflammatory activation phenotype from the proinflammatory ("M1-like") to the anti-inflammatory and immunoregulatory ("M2-like") phenotype. Our results indeed show that IRF3-overexpressing microglia upregulate key anti-inflammatory cytokines and downregulate proinflammatory cytokines such as IL-1. We provide evidence that the PI3K/Akt pathway plays an anti-inflammatory role in microglia and that IRF3-mediated microglial phenotype switch is associated with augmentation of Akt activation.

## List of Abbreviations

Ad-GFP: adenovirus-green fluorescence protein; Ad-IRF3: adenovirus-IRF3; Akt: = PKB: protein kinase B; CXCL1: CXC chemokine ligand 1 = GROα; GAPDH: glyceraldehydes 3-phosphate dehydrogenase; IFIT1: Interferon-induced protein with tetratricopeptide repeats 1; IFNλ: interferon lambda, type III interferon; IL-1ra: IL-1 receptor antagonist; *IL-1RN *(human IL-1 receptor antagonist gene; iNOS: inducible nitric oxide synthase; IRF3: interferon regulatory factor 3; MAPK: MAP kinase; miR-155: microRNA-155; MyD88: myeloid differentiation primary response gene 88; M1: macrophages classical activation phenotype; M2: macrophage alternative activation phenotype; PBDA: porphobilinogen deaminase; PIC: poly IC; PTEN: phosphatase and tensin homologue; Q-PCR: real-time reverse transcription PCR; SOCS1: suppressor of cytokine signaling 1; TRIF: TIR domain-containing adaptor inducing IFNβ.

## Competing interests

The authors declare that they have no competing interests.

## Authors' contributions

LT and HS performed the experiments and interpreted the data; LT and SCL designed the experiments, analyzed the data and wrote the paper. All authors read and approved the final version of the manuscript.

## Supplementary Material

Additional file 1 **Microarray studies of microglia transduced with Ad-IRF3 or Ad-β-gal and stimulated with poly IC (PIC)**. Complete data set of microarray of microglia as shown in Table 1. Click here for file

Additional file 2 **Microarray studies of microglia transduced with Ad-IRF3 or Ad-GFP and stimulated with IL-1/IFNγ with or without LY294002**. Complete data set of microarray of microglia as shown in Table 2 and Figure 6A. Click here for file
